# Trastuzumab in advanced breast cancer – a decade of experience in Germany

**DOI:** 10.1186/1471-2407-14-924

**Published:** 2014-12-08

**Authors:** Christian Jackisch, Winfried Schoenegg, Dietmar Reichert, Manfred Welslau, Johannes Selbach, Hanns-Detlev Harich, Hans Tesch, Tim Wohlfarth, Heidi Eustermann, Axel Hinke

**Affiliations:** Department of Obstetrics and Gynaecology and Breast Cancer Center, Sana Klinikum Offenbach GmbH, Starkenburgring 66, D-63060 Offenbach, Germany; Practice, Berlin, Germany; Practice, Westerstede, Germany; Practice, Aschaffenburg, Germany; Practice, Duisburg, Germany; Practice, Hof, Germany; Hämatologisch-Onkologische Gemeinschaftspraxis am Bethanien-Krankenhaus, Frankfurt am Main, Germany; Roche Pharma AG, Grenzach-Wyhlen, Germany; WiSP Research Institute, Langenfeld, Germany

**Keywords:** HER2 overexpression, Trastuzumab, Advanced breast cancer, Non-interventional study, Elderly patients

## Abstract

**Background:**

Trastuzumab was registered in 2000 for the treatment of metastatic breast cancer, both as monotherapy and combination therapy with paclitaxel. In this prospective, non-interventional observation study, the 10-year experience with trastuzumab in the routine management of HER2-positive breast cancer was reviewed.

**Methods:**

Between 2000 and 2010, 1843 evaluable patients with advanced HER2-positive breast cancer were recruited in 223 institutions across Germany. Patients were prospectively monitored for about one year. Additional information on long-term outcomes, progression-free survival (PFS), and overall survival (OS) were retrieved at several follow-up points. There were no restrictions with respect to diagnostic or therapeutic procedures. Patients were stratified into three cohorts depending on the treatment regimen, i.e. trastuzumab monotherapy (n =228, 12%), trastuzumab combined with chemotherapy (n =1346, 73%), or trastuzumab combined with endocrine therapy (n =269, 15%).

**Results:**

Median age was 59.5 years with a proportion of 28% being older than 65 years. Over a maximum follow-up period of more than 10 years, 1538 PFS events were documented in 83% of patients, resulting in an estimated median PFS of 11.8 months. Median OS, based on recorded death in 64% of patients, amounted to 34.4 months, with 48% (95% confidence intervals 45 – 50%) still alive after three years. The subgroup selected for a treatment combination with endocrine drugs only had distinctly longer PFS and OS than the other two groups, achieving medians of 23.3 months and 56.3 months, respectively. Median PFS and OS in elderly patients over 65 years of age was 11.4 months and 28.3 months, respectively. Adverse reactions, including cardiac toxicity, of severity grade 3 or 4 were rare.

**Conclusions:**

The superior outcome of treatment strategies including trastuzumab in HER2 overexpressing breast cancer, proven in pivotal studies, was confirmed in the management of advanced breast cancer in Germany in the routine setting. Our data suggest a comparable clinical benefit of treatment with trastuzumab in elderly patients (>65 years), who are typically under-represented in randomized clinical studies.

## Background

Trastuzumab (Herceptin®) was registered in Germany in 2000 for the treatment of HER2-positive metastatic breast cancer (MBC), either as single agent in pretreated patients or as first-line therapy in combination with paclitaxel. The latter was based on a pivotal trial demonstrating that the addition of the humanized antibody, trastuzumab, to taxane led to improved clinical outcomes including longer OS, compared with single-agent paclitaxel, despite a crossover rate of approximately 70% [1]. As a result of subsequent phase III trials [2, 3], trastuzumab was registered in 2004 for use in combination with docetaxel and in 2007 for use with aromatase inhibitors. Today, trastuzumab-based therapy is considered the standard of care for adjuvant or palliative treatment of HER2-positive breast cancer [4, 5].

This observational study comprising almost 2000 patients reflects the full spectrum of trastuzumab use in routine practice in metastatic or locally advanced breast cancer (LABC), with a patient population distinctly different from that typically recruited in phase III clinical trials, particularly with respect to age. Our objective was to assess patient characteristics and patterns of care during a period of 10 years, and to compare the long-term results with those achieved in the pivotal trials.

## Methods

### Patient population and methods of observation

This non-interventional observation study focused on patients with advanced breast cancer (MBC or LABC), fulfilling the selection criteria according to the registered drug label for trastuzumab (Herceptin®) in Germany. All types of pretreatments were acceptable. HER2 positivity was usually defined as 3+ staining in immunochemistry or a positive result of fluorescence in situ hybridization (FISH) in case of 2+ staining. Patients were treated in accordance with the routine practice of the respective institution, and findings were prospectively documented on standardized case report forms. There were no restrictions with respect to individual diagnostic and therapeutic procedures after patient registration, namely concerning the concurrent administration of other antineoplastic agents. The patients’ course of disease and treatment were closely monitored through data queries, either until trastuzumab therapy stop for whatever reason, or for a treatment period of at least 12 months. Thereafter, key long-term data were regularly retrieved by fax forms until the patient’s death. Adverse drug reactions (ADR), as defined in the case report form, were recorded according to the regulations of the German drug law. Physicians from hospitals or practices were invited to participate, either during the whole study period or only for parts of it. Database closure was September 2012.

This was an observational study in which physicians’ choices were guided by drug registration status and treatment guidelines (rather than the observation protocol). As the study was started prior to 2007, it was in agreement with the German FSA Codex [6] and the German Arzneimittelgesetz Amendment 12, there was no need/requirement for ethics committee approval or written informed consent. For non-interventional studies started in 2007 or later, the FSA Codex asks for submission to the ethics committee and to the regulators. Furthermore, in the European Union, clinical research has to be performed according to the Directive 2001/20/EC of the European Parliament and of the Council on the approximation of the laws, regulations and administrative provisions of the Member States relating to the implementation of good clinical practice in the conduct of clinical trials on medicinal products for human use dating from April 2001. This regulation differentiates between the requirements for “interventional” and “non-interventional” studies. This observational study clearly fulfills the criteria for “non-interventional” as defined in Article 2, c.

### Endpoint evaluation and statistical aspects

Tumor regression and progressive disease (PD) was recorded as the best response achieved, based on standard clinical procedures at the discretion of the investigators, without formal requirement of objective remission confirmation. PFS and OS were calculated as the time from the first trastuzumab administration to the respective event. Surviving patients without PD were censored at the last valid observation point. Safety data were collected during the 12-month period of detailed documentation, but events reported afterwards were also included in the analysis.

Event-related endpoints were analyzed using the Kaplan-Meier method, providing 95% confidence intervals (CIs) for proportions at specific time points. Univariate analysis of potential prognostic factors was performed using the logrank test [7]. All prognostic factors with an associated *P* value <0.1 in the univariate analysis were included in a multivariate Cox proportional hazards model [8]. By backward selection, all ‘unnecessary’ variables were removed step-by-step, so that the final model only contained covariates with a *P* value ≤0.05. Hazard ratios (HR) with 95% CIs were retrieved from this model. Examination of the treatment decision process was performed using standard contingency table methods and logistic regression. All statistical analyses were of exploratory nature, with no adjustment of *P* values for multiplicity. The term “significant” was used in case of *P* ≤0.05. All reported *P* values are two-sided.

## Results

Overall, 1914 documentation forms were obtained from 223 clinics and practices across Germany between 2000 and 2010. After exclusion of clearly ineligible cases (mostly patient reports referring to adjuvant trastuzumab treatment), 1843 patients with advanced HER2-positive breast cancer remained for this analysis. Although trastuzumab was only approved for the treatment of metastatic breast cancer at the time recruitment started, 10% of patients suffered from non-metastatic, locally recurrent disease. Most patients (1346; 73%) received the first trastuzumab-based therapy along with cytotoxic treatment. Overall, 269 (15%) patients received the antibody in combination with endocrine therapy, while the remaining 228 (12%) patients received trastuzumab monotherapy. Most results are presented separately for these subgroups.

### Baseline characteristics

Table 1 shows the patient and tumor characteristics before start of trastuzumab treatment. A considerable number of patients were elderly, with the proportion of participants ≥65 years of age increasing from 27% in the first four years of recruitment to 40% thereafter. In general, patients treated with trastuzumab in combination with endocrine therapy were older and showed a more favorable prognostic profile, i.e. a better performance status, less G3 tumors, a longer relapse-free interval, fewer metastatic sites, a focus on bone rather than visceral disease, less palliative pretreatment, and a positive hormone receptor status. Women receiving trastuzumab monotherapy were typically more heavily pretreated with palliative chemotherapy. In this subgroup, 28% of patients had previously undergone one regimen for advanced disease, while 11% of patients had received two and 14% of patients three or more previous regimens for advanced disease. No differences between treatment groups with respect to baseline cardiac function were reported.

**Table 1:** **Patient and tumor characteristics (n =1843)**

Parameter		Treatment		
	Trastuzumab monotherapy	Trastuzumab plus chemotherapy	Trastuzumab plus endocrine therapy only	Total
**Number of patients**	228 (12%)	1346 (73%)	269 (15%)	1843 (100%)
**Age**				
Median (range) [years]	59.8 (31 – 91)	58.8 (21 – 87)	61.8 (31 – 95)	59.5 (21 – 95)
>65 years	26%	26%	36%	28%
>70 years	16%	13%	20%	14%
**ECOG performance status**				
0	29%	30%	37%	31%
1	56%	55%	51%	54%
2	13%	13%	11%	13%
3-4	2%	2%	1%	2%
**Tumor grade**				
G1	2%	3%	5%	3%
G2	49%	42%	49%	44%
G3	49%	55%	46%	53%
**M1 disease at primary diagnosis**	24%	26%	28%	26%
**Relapse-free interval, median [years]**	2.0	2.2	2.6	2.2
Hormone receptor status*				
Estrogen-receptor positive	41%	54%	84%	57%
Progesterone-receptor positive	34%	47%	68%	48%
At least one positive	44%	58%	87%	61%
**Metastatic sites at onset of trastuzumab treatment**				
0	18%	7%	14%	10%
1	45%	42%	52%	44%
2	25%	33%	23%	31%
3	8%	13%	9%	12%
≥4	4%	5%	1%	4%
**Organ site involvement**				
Liver	34%	45%	25%	41%
Lung	26%	34%	21%	31%
Bone	41%	45%	55%	46%
Central nervous system	8%	5%	2%	5%
Pleural effusion	9%	12%	9%	11%
Ascites	1%	2%	1%	1%
Other	20%	20%	19%	20%
**Previous treatment**				
Radiotherapy	68%	63%	67%	64%
Adjuvant chemotherapy	62%	61%	54%	60%
Adjuvant endocrine therapy	30%	42%	60%	43%
Palliative chemotherapy	52%	36%	35%	38%
Palliative endocrine therapy	19%	28%	51%	30%
Received anthracycline and taxane	48%	37%	46%	40%
Received trastuzumab	20%	7%	13%	9%
No. of previous palliative chemotherapy regimens (n =692**)				
1	53%	56%	63%	57%
2	20%	24%	21%	23%
3	14%	10%	9%	11%
4	12%	8%	5%	8%
**LVEF, median (range) [%]**	65 (30 – 82)	65 (35 – 95)	65 (40 – 98)	65 (30 – 98)

* unknown in 5% of patients, ** population with palliative cytotoxic pretreatment.

*Abbreviations*: *ECOG* Eastern Cooperative Oncology Group, *LVEF* Left ventricular ejection fraction.

### Treatment

In line with the limited period of detailed data recording, median duration of documented trastuzumab treatment amounted to almost exactly one year, since half of the patients were reported to be treated for more than 52 weeks. However, median duration of the antibody therapy without detection of tumor progression was 43 weeks only, indicating a trastuzumab treatment in multiple lines in a considerable number of patients (see below). When including the follow-up information received via fax transmission, median treatment duration rose to 64 weeks overall (55 weeks in the monotherapy subgroup, 62 weeks in the chemotherapy subgroup, and 98 weeks in the endocrine therapy subgroup). In total, more than one third of the patients received trastuzumab for more than two years. As the three-weekly schedule became an alternative option to the initially approved weekly application only late during the observation study period, 64% of the patients received 2 mg/kg body weight, and 28% of patients received 6 mg/kg. (Due to the loading-dose strategy, these figures are based on analysis of the second trastuzumab application).

Among the 1336 patients for whom the concomitant cytotoxic regimen was known, 78% received only one cytotoxic agent. Almost half of the patients (47%) received a taxane, predominantly paclitaxel. The other chemotherapeutics frequently combined with trastuzumab were vinorelbine (23%) and capecitabine (6%). Anthracyclines were administered concurrently with trastuzumab in about 4% of the patients in the chemotherapy subgroup.

The reasons for using trastuzumab in combination with cytotoxic agents were studied in further detail. In the univariate analysis, age ≤65 years (P =0.033), negative hormone receptor status (P =0.0012), two or more sites of metastasis (P <0.0001), and visceral metastasis (P <0.0001) were significantly associated with the decision to administer chemotherapy together with trastuzumab. In contrast, the relapse-free interval, stage IV disease at presentation, and CNS metastases had no major impact on this decision. In a multivariate logistic regression model, hormone receptor status (P =0.00064) and visceral metastases (P <0.0001) remained highly significant independent predictors.

### Efficacy

In the 1737 patients evaluable for response, complete remission (CR) was reported in 263 (15%) patients and partial remission (PR) in 743 (43%) patients. A further 523 (30%) patients experienced stable disease, whereas 12% showed signs of primary PD. This resulted in an overall response rate (ORR) of 58% (95% CIs 56 to 60%). ORR was highest in the subgroup receiving trastuzumab together with chemotherapy (60%). In the subgroups receiving trastuzumab monotherapy or trastuzumab combined with endocrine treatment, ORRs amounted to 44% and 40%, respectively. In patients with chemotherapeutic pre-treatment for advanced disease, ORR was lower (53%). The same holds true for patients having previously received both anthracyclines and taxanes (adjuvant or palliative; ORR =51%).

**Figure 1:**
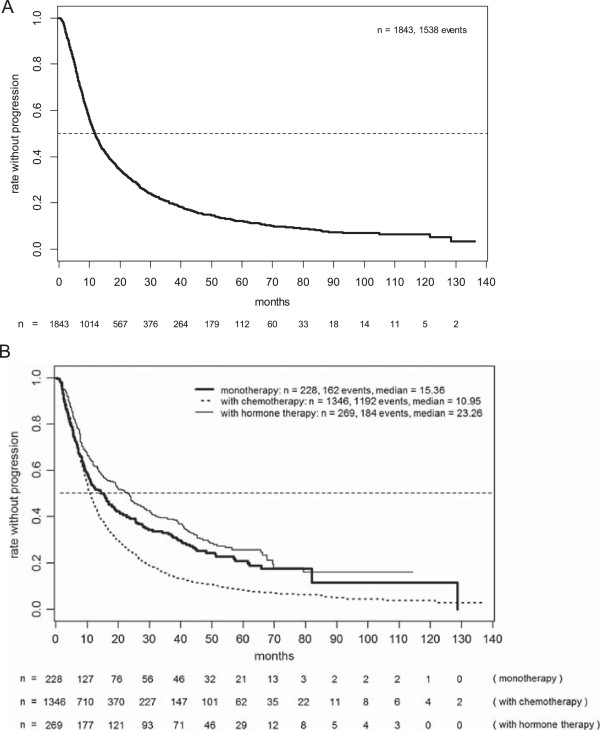
**Progression-free survival in the total patient population (A) and the various subgroups (B).**

So far, 1538 PFS events (83%) and 1174 deaths (64%) have been recorded in the database, with a maximum follow-up period of more than 10 years. Figure 1A shows PFS for the whole study population (median: 11.8 months, 95% CIs 11.1 to 12.6 months), and Figure 1B for the treatment-based subgroups with monotherapy (median: 15.4%), chemotherapy (11.0 months), and endocrine therapy (23.3 months), clearly documenting the rationale of treatment choice depending on prognostic factors. After two years, PFS rates were 30% overall (95% CIs 28 to 32%), and 39%, 25%, and 49% in the respective subgroups. There were no major differences with respect to median PFS depending on the type of concomitant chemotherapy chosen (11.5 and 10.8 months for taxane and vinorelbine, respectively, and 10.3 months in patients selected for polychemotherapy).

**Figure 2:**
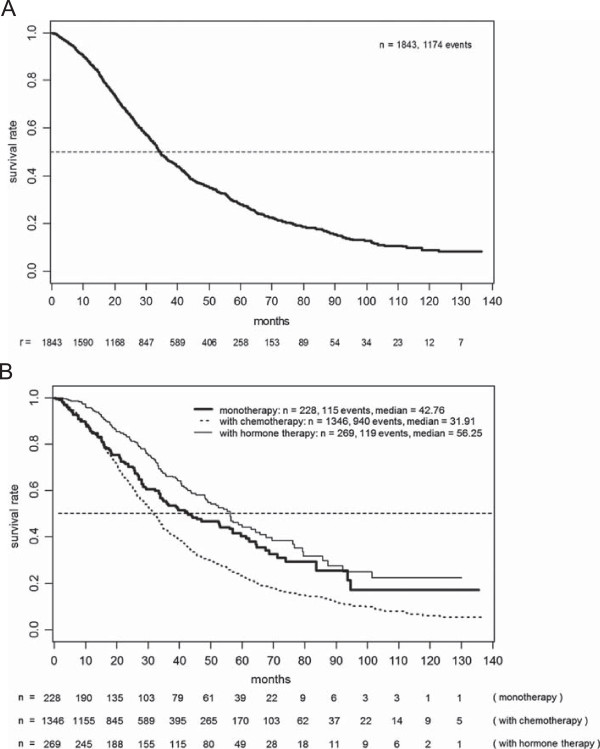
**Overall survival in the total patient population (A) and the various subgroups (B).**

Figure 2 shows OS based on 1174 (64%) reported deaths for the whole population and the subpopulations. Overall median survival amounted to 34.4 months (95% CIs 33.2 to 36.1 months), with 48% (95% CIs 45 to 50%) still living after three years. Because of the criteria applied when selecting the patients’ treatment, median survival was considerably shorter in patients simultaneously treated with chemotherapy (31.9 months) than those undergoing monotherapy with trastuzumab (42.8 months) or those receiving trastuzumab combined with endocrine therapy (56.3 months). Three-year survival rates were 43%, 55%, and 66%, respectively.

### Prognostic factors for long-term results

The impact of several prognostic characteristics on PFS and OS was analyzed, focusing on the subgroup of patients receiving trastuzumab in combination with chemotherapy, in order to achieve homogeneity and avoid interactions between baseline factors and treatment decision (Table 2). PFS was significantly longer in patients without previous chemotherapy for advanced disease (median, 11.8 vs. 9.5 months), with bone-only metastases (13.9 vs. 10.2 months), and with stage IV disease at presentation (13.1 vs. 10.1 months). No major prognostic impact was detected for hormone receptor status or age, with medians of 10.7 months and 11.4 months in the cohorts aged ≤65 years and >65 years, respectively. In a multivariate Cox model, bones as the sole metastatic site and stage IV at presentation remained the only independent significant factors.

**Table 2:** **Univariate and multivariate analysis of prognostic factors for progression-free survival and overall survival**

Parameter	Progression-free survival		Overall survival	
	Univariate p	Multivariate p	Univariate p	Multivariate p
Palliative cytotoxic pretreatment	0.00051	--	< 0.0001	--
Age >65 years	--	--	0.072	0.038
Metastases other than skeletal	0.0061	0.032	0.0041	0.016
Hormone-receptor negative	--	--	0.039	--
M0 at initial diagnosis	0.0071	0.0013	0.040	0.0046

-- denotes p >0.1.

With respect to OS, previous cytotoxic therapy for advanced disease (median, 27.4 vs. 34.6 months), age >65 years (28.3 vs. 33.4 months), bone lesions only (41.0 vs. 30.0 months), hormone receptor positivity (33.2 vs. 29.4 months), and stage IV at presentation (34.8 vs. 31.4 months) showed a correlation of at least borderline significance. In the regression model, age, bone lesions only, and stage IV disease retained the conventional significance level.

Among the total patient population, 90 patients with CNS metastases were identified, exhibiting distinctly shorter PFS (median, 7.5 vs. 12.0 months, *P* <0.0001) and OS (median, 20.3 vs. 34.8 months, *P* <0.0001).

### Trastuzumab treatment beyond progression

Among the patients entering the study while undergoing first-line treatment for advanced disease, 418 women fulfilled the criteria for an analysis of treatment beyond progression and its impact on the course of disease. Both univariate and multivariate analyses suggested distinctly longer survival in the 261 patients with continued trastuzumab treatment. These results are presented in a separate publication [9].

### Safety

Trastuzumab was well tolerated with predictable and manageable ADR both when given as monotherapy and in combination with other treatments. Table 3 presents the National Cancer Institute Common Toxicity Criteria [NCI CTC] grade 3/4 ADR with an incidence of ≥1% in the total population by subgroups. The most common grade 3/4 ADR was leukopenia with a frequency of 5%, but this was only observed in the chemotherapy subgroup. Cardiac toxicity occurred with an incidence of 2.3% across all severity grades. Grade 3 ADR occurred in 0.5% (no grade 4 event). However, this proportion was distinctly lower in patients aged <65 years than in the older patients (1.5% vs. 4.2%).

**Table 3:** **Frequency of adverse drug reactions of grade 3/4 severity (highest NCI CTC grade per category and patient)**

Adverse event/organ system	Patients with NCI CTC grade [n (%)]			
	T*	T + CT**	T + HT***	Total
**Hematological**				
Hemoglobin decreased	-	13 (1%)	-	13 (1%)
WBC decreased	1 (0%)	57 (4%)	-	58 (3%)
Granulocytes decreased	1 (0%)	16 (1%)	1 (0%)	18 (1%)
**Non-hematological**				
Dyspnea	1 (0%)	18 (1%)	1 (0%)	20 (1%)
Pain	2 (1%)	16 (1%)	3 (1%)	21 (1%)

*trastuzumab monotherapy, **trastuzumab plus chemotherapy, ***trastuzumab plus endocrine therapy only.

NCI CTC: National Cancer Institute Common Toxicity Criteria; WBC: white blood cells.

## Discussion and conclusions

This observation study evaluated the use of trastuzumab in advanced HER2-positive breast cancer since its registration in 2000, based on the experience in a representative selection of more than 200 clinics and practices in Germany outside the setting of a prospective interventional clinical trial. To the best of our knowledge, our data represents information on the longest follow-up period reported on trastuzumab treatment in this setting. Moreover, the study provides important data on the use, efficacy, and safety of trastuzumab under “real-life” conditions in a large patient cohort.

When comparing our data with those obtained from the pivotal studies that typically involve selected target groups, a striking difference with respect to age distribution is apparent. In the registration study by Slamon et al. [1], mean and median age was 53 years, with mean ages in subsequent randomized studies ranging from 54 to 56 years [2, 3, 10, 11]. Thus, the patients participating in the randomized trials were considerably younger than those assessed in the present study (median age of almost 60 years). Even the French HERMINE study that retrospectively selected a cohort from 2002, included patients with a lower median age, i.e. 54 years [12]. Similarly, only 21% of the 1001 patients participating in the US-based observational registHER study between 2003 and 2006, were beyond the age of 65 years, as reported in a recent publication focusing on elderly patients [13].The increasing numbers of elderly patients treated with trastuzumab in more recent years is thought to be the result of the growing clinical experience with the use of this antibody. In our study, the proportion of patients aged 65 years or more increased from 27% (by 2003) to 39% in the period thereafter.

Although earlier clinical trials and the present observational study differ in a number of respects, our results confirm the favorable outcomes reported in the pivotal studies. The high ORR may partly be due to some limitations with respect to defined response criteria and requirement of remission confirmation. However, the median PFS of almost one year in the overall population and the chemotherapy/trastuzumab subgroup compares well with data published from interventional studies on taxane/trastuzumab regimens [14]. In the paclitaxel subgroup of the initial registration study (HO648g) by Slamon et al., the lower median PFS of 6.9 months may be explained by the inclusion of patients with HER2 overexpression of 2+ only [1]. The more recent studies involving trastuzumab combined with docetaxel [2, 11] reported PFS values of 11.7 and 12.4 months, respectively, which are very similar to ours. The same applies to the recently published FAKT study involving weekly treatment with paclitaxel combined with trastuzumab [15].

Translation of the beneficial results from clinical trials into routine practice is even more convincingly shown with respect to OS where a median of about 34.5 months (both in the total population and the chemotherapy/trastuzumab subgroup) was achieved. Again, in the HO648g study, median OS was somewhat lower (25.1 months), but our results are in good agreement with medians for combinations with docetaxel (31.2 months and 35.7 months) and vinorelbine (38.8 months) [2, 11]. Nevertheless, when comparing our results to the pivotal studies, one clearly has to acknowledge the limitation of a possible data collection bias. In particular, we have no access to data from those patients, which were not assigned to trastuzumab treatment in spite of a positive HER2 status. Moreover, the selection process has certainly changed a lot during the ten year study period.

In MBC the additional option of using a dual blockade in HER2 overexpressing endocrine sensitive breast cancer (Her2+/ER+) was not widely implemented in the routine setting in Germany during our study period. The combination of endocrine therapy plus trastuzumab without chemotherapy was used in 15%, only. Interestingly this option was widely used in the elderly women (Table 1). However, in our routine setting, the risk-adapted selection of patients for endocrine therapy in combination with trastuzumab resulted in an exceptionally long median OS of almost 5 years. The importance of the correct patient selection was clearly shown in the two randomized studies with trastuzumab/endocrine drug combinations, for which distinctly different PFS results (i.e. 4.8 months and 14.1 months) were reported [3, 16]. In summary, the option to combine aromatase inhibitors either with trastuzumab or lapatinib in those individuals not being an ideal candidate for a chemotherapy bases regime seems to be a perfect and well tolerated option in controlling this subtype of MBC.

Likewise, the favorable long-term data obtained for the trastuzumab monotherapy group in our observational study contrasts with published data on trastuzumab monotherapy in the US or German compassionate-use trials. There, PFS medians of only 3 to 5 months were achieved [17–19]. Thus, the careful selection of patients with a relatively low metastatic burden or even locoregional disease, in our cohort appears to be responsible for the favorable outcome in this subgroup.

The outcome of trastuzumab treatment in elderly patients with advanced breast cancer has been specifically addressed in the registHER study [13]. Both their and our data show no inferior results for patients ≥65 vs. <65 years in median PFS (11.7 vs. 11.0 months, and 11.4 vs. 10.7 months, respectively). However, the corresponding OS data show some difference, again uniformly in both studies, with 31.2 vs. 40.4 months, and 28.3 vs. 33.4 months, respectively. This may be due, in part, to more deaths not related to breast cancer in the older patient group. As described elsewhere [9], we were able to analyze trastuzumab treatment beyond progression in a rather large subpopulation (n = 418), confirming the favorable outcome reported in the randomized study [20].

It is of special importance that despite the large number of patients included in the present observational study, no major new safety issues emerged. The low frequency of ADR points to an underreporting, which is a clear limitation of the observational study design. In the subgroup receiving trastuzumab combined with chemotherapy, toxic effects were more likely assigned to the chemotherapy than to trastuzumab. Significant cardiac problems occurred very rarely, albeit with an expected higher frequency in elderly patients.

Recent developments have greatly expanded the armamentarium of drugs targeting HER2-positive breast cancer [21]. This includes the pharmacokinetically bioequivalent option of subcutaneous administration of trastuzumab, which is strongly preferred by the patients [22], and a first antibody-cytotoxic conjugate, emtansine, highly active after trastuzumab pre-treatment [23, 24]. Moreover, the tyrosine kinase inhibitor lapatinib in second-line combinations, namely with simultaneous trastuzumab [25], and the synergistically efficacious combination of trastuzumab and pertuzumab [26] constitute valuable alternatives. These findings confirm, for the time being, trastuzumab remains the essential cornerstone of any routine treatment strategy in HER2-positive breast cancer.
